# Clotting Factor Deficiencies as an Underlying Cause of Abnormal Uterine Bleeding in Women of Reproductive Age: A Literature Review

**DOI:** 10.3390/life13061321

**Published:** 2023-06-05

**Authors:** Maria Effrosyni Livanou, Alkis Matsas, Serena Valsami, Dimitrios T. Papadimitriou, Athanasios Kontogiannis, Panagiotis Christopoulos

**Affiliations:** 1 Second Department of Obstetrics and Gynecology, “Aretaieion” Hospital, Faculty of Medicine, National and Kapodistrian University of Athens, 115 28 Athens, Greece; 2 Hematology Laboratory-Blood Bank, Aretaieion Hospital, National and Kapodistrian University of Athens, 115 28 Athens, Greece

**Keywords:** Abnormal Uterine Bleeding, Heavy Menstrual Bleeding, menstruation, coagulopathy, clotting factor deficiency, hemophilia carriers

## Abstract

Clotting Factor deficiencies are rare disorders with variations in clinical presentation and severity of symptoms ranging from asymptomatic to mild to life-threatening bleeding. Thus, they pose a diagnostic and therapeutic challenge, mainly for the primary health care providers, general practitioners, and gynecologists who are more likely to first encounter these patients. An additional diagnostic challenge arises from the variable laboratory presentations, as PT, PTT, and BT are not always affected. The morbidity is higher among women of reproductive age since Abnormal Uterine Bleeding–specifically Heavy Menstrual Bleeding–is one of the most prevalent manifestations of these disorders, and in some cases of severe deficiencies has led to life-threatening episodes of bleeding requiring blood transfusions or even immediate surgical intervention. Physician awareness is important as, in the case of some of these disorders–i.e., Factor XIII deficiency–prophylactic treatment is available and recommended. Although uncommon, the potential for rare bleeding disorders and for hemophilia carrier states should be considered in women with HMB, after more prevalent causes have been excluded. Currently, there is no consensus on the management of women in these instances and it is reliant on the physicians’ knowledge.

## 1. Introduction

Menstrual problems are common in adolescent women and women of reproductive age in general [1,2]. According to the bibliography, up to 10–35% of women report Heavy Menstrual Bleeding (HMB) at some point during their reproductive lives [1]. The causes of HMB are currently classified according to the PALM-COEIN system of nomenclature. According to this system, they are divided into structural causes (Polyp, Adenomyosis, Leiomyoma, Malignancy, and Hyperplasia) and functional causes (Coagulopathy, Ovulatory Disorder, Endometrial, Iatrogenic, Not Yet Classified) [2]. Anovulation is likely the most common cause of HMB, at least in the adolescent age group. However, the published literature shows that 10–62% of adolescents with HMB may have an underlying Inherited Bleeding Disorder (IBD) - Coagulopathy [1]. Vice versa, a common initial presentation for women with an underlying coagulation disorder is Heavy Menstrual Bleeding (HMB).

The most common bleeding disorder is von Willebrand disease, accounting for 5% to 20% of HMB cases [3]. Since Von Willenbrand disease has been extensively investigated, it is not discussed in this review. However, less common bleeding disorders have not received the same amount of attention, although they can be a cause of severe morbidity for women of reproductive age. In this review, our goal was to focus on the rest of the secondary hemostasis disorders included in the Coagulopathy category: Rare Coagulation Factor Deficiencies (RCFDs) and Hemophilia Carriers. 

Rare Coagulation Factor Deficiencies is an umbrella term used to describe autosomal recessive disorders which are caused by the deficient production or activity of a clotting factor, excluding factor XII and hemophilia factors VIII and IX. They have been reported to account for approximately 3–5% of bleeding disorders, although data from a 2016 survey by the World Federation of Hemophilia and the Rare Bleeding Disorders Database suggest that the prevalence might be as high as 8%. It is estimated that severe forms of these disorders affect anywhere from 1 person in 500,000 up to 1 person in 2–3,000,000, according to the defect. Diagnosis is based on past medical and family history of bleeding, coagulation tests, and factor assays. Genetic diagnosis may be helpful to determine the bleeding phenotype, as there is no linear relationship between bleeding severity and clotting factor levels [4].

Hemophilia Carriers are female carriers of X-linked disorders of factors VIII (Hemophilia A) or IX (Hemophilia B). Historically females were considered to be asymptomatic in contrast to men. However, this view has recently changed as accumulating evidence indicates that up to 40–60% of hemophilia carriers may experience clinically significant bleeding. HMB is one of the clinical challenges these women face, and it will be specifically addressed in this review [5].

All factor deficiencies share similar clinical and laboratory features. In women of reproductive age, they usually manifest with Abnormal Uterine Bleeding, while more serious presentations include ovulation-induced hemoperitoneum and postpartum hemorrhage. In some patients, factor deficiencies may also cause mucosal bleeding, gingival bleeding, rhinorrhagia-ecchymoses, epistaxis, gastrointestinal (GI) bleeding, and hemorrhage after minor trauma. The relative frequency and the severity of each bleeding manifestation differs depending on the specific factor deficiency and the severity of the deficiency. In severe deficiencies, serious hemorrhagic events, such as intracerebral hemorrhage and life-threatening bleeding following trauma, have been reported. Common laboratory findings include prolonged Prothrombin time (PT) and Partial Thromboplastin time (PTT), which correct after mixing [5,6,7,8,9]. Mixing studies can distinguish if the PT or PTT elevation is caused by a factor deficiency or a factor inhibitor. It is performed by mixing plasma from the patient with control plasma. If PT and PTT correct after mixing, then the underlying pathology is a factor deficiency [10].

Successful management and prevention of gynecological complications in women with clotting factor deficiencies requires a collaboration between hemophilia and gynecology teams. The aim of HMB management is to prevent serious bleeding episodes, replete iron storage, ameliorate quality of life, and reduce morbidity. Recommendations regarding the management of other bleeding events and surgeries in these women rely on the published literature and expert opinions [11]. In acute bleeding settings, the need for hemostatic treatment is determined by the remaining factor levels, the severity and location of bleeding, and the history of previous bleeding events.

In the next sections, we refer individually to Rare Coagulation Factor Deficiencies (namely Factor I, V, VII, Combined Factor V and VIII, X, XI, and XIII deficiencies) as well as hemophilia A and B carriers (factors VIII and IX) and their associations with Abnormal Uterine Bleeding, including the frequency and severity of this clinical manifestation in women of reproductive age, as well as the management strategies that are being used. A summary of evidence about the estimated prevalence of each of these disorders and the respective incidence of HMB can be found in Table 1.

## 2. Discussion

### 2.1. Factor I (Fibrinogen) Disorders

Fibrinogen (Factor I) is a soluble plasma glycoprotein which is synthesized in the liver and secreted in the plasma. It has a crucial role in the final step of the coagulation cascade and is also implicated in inflammation and wound healing [12,25]. Three genes, the fibrinogen alpha chain (*FGA*), fibrinogen beta chain (*FGB*), and fibrinogen gamma chain (*FGG*) gene, located on chromosome 4q23.5, encode for the three peptide chains which form fibrinogen. Mutations in these genes cause congenital fibrinogen defects [12].

Patients with congenital afibrinogenemia lack plasma fibrinogen. The disease is inherited in an autosomal recessive manner and is due to mutations in the FGA gene, leading to disruptions in protein synthesis, assembly, or secretion. Like most other coagulation factor deficiencies, it is very rare, having an estimated prevalence of 1 in 1,000,000 [12,13]. However, afibrinogenemia is considered to be more common in communities where consanguineous marriages are common such as Iran [14]. Patients with afibrinogenemia have very low or undetectable fibrinogen levels and clinical manifestations range from minimal bleeding to serious hemorrhages [12,13]. Paradoxically, patients are also prone to arterial or venous thrombosis [13,25]. PT, PTT, and TT are infinitely prolonged, as they are all parameters depending on the formation of fibrin [26].

Patients with hypofibrinogenemia, on the other hand, have reduced amounts of circulating functional fibrinogen. The deficiency can be mild, moderate, or severe. The majority of these patients are carriers of a single copy of an afibrinogenemia mutation, although there have been occasional reports of missense variants [12,13]. In cases of hypofibrinogenemia, the levels of functional and antigenic fibrinogen are proportionally reduced. Patients with fibrinogen levels above 1 g/L are typically asymptomatic. However, decreased levels of functional fibrinogen may occasionally lead to a low tendency for bleeding or, less commonly, thrombotic complications [13]. In affected individuals, PT, PTT, and TT are variably prolonged, with TT being the most sensitive assay [26].

Congenital dysfibrinogenemia is characterized by abnormal structure and function of the fibrinogen molecules. Despite having normal antigen levels, these patients experience decreased functional activity. The inheritance pattern is typically autosomal dominant [12,13]. Most individuals with dysfibrinogenemia do not exhibit symptoms or have mild symptoms, resulting in incidental diagnosis or detection during presurgical hemostasis evaluations. However, some patients may experience significant bleeding symptoms and develop venous or arterial thrombosis [13]. The thrombin time (TT) may be prolonged in certain forms of dysfibrinogenemia, although it can also be within the normal range [26]. Hypodysfibrinogenemia is characterized by reduced levels of a malfunctioning fibrinogen molecule. It is further categorized into three subtypes based on the antigen level of fibrinogen. Patients with hypodysfibrinogenemia may either exhibit no symptoms or experience episodes of bleeding and/or thrombosis [12,13]. Determining the accurate prevalence of dysfibrinogenemia and hypofibrinogenemia is challenging due to a large number of asymptomatic individuals [13]. Congenital fibrinogen disorders account for 7–10% of Rare Clotting Factor Deficiencies [11]. Diagnosis is confirmed by showing reduced activity and/or reduced levels of immunoreactive FIB in the plasma [25].

Patients with severe congenital afibrinogenemia typically exhibit an early onset of clinical symptoms. The first bleeding episode in these patients is often umbilical cord hemorrhage or prolonged umbilical stump bleeding [25]. Other symptoms that affect people with congenital fibrinogen disorders are instances of mucocutaneous bleeding such as ecchymotic patches, epistaxis, and gum bleeding [15]. Nevertheless, menorrhagia is recognized as the predominant, and in some cases, the sole symptom experienced by patients with congenital fibrinogen disorders [15,25]. Data regarding the prevalence of AUB/menorrhagia in women with CFD are depicted in Table 2 (only female patients after the onset of menarche are included).

In cases of severe bleeding episodes, replacement therapy remains the most effective and sometimes the only available option. Fibrinogen replacement products encompass fresh frozen plasma (FFP), cryoprecipitates, and plasma-derived fibrinogen concentrates. Among these three options, plasma-derived fibrinogen concentrates are considered the optimal choice due to their superior safety profile compared to the other two alternatives, as they minimize the risk of viral infection and fluid overload. It is strongly advised to avoid the use of whole blood transfusion, particularly in young women, to prevent potential allergic reactions [25]. When addressing menorrhagia in congenital fibrinogen disorders, commonly employed therapeutic products include FFP, cryoprecipitate, and antifibrinolytics [15]. Additional treatments such as hormonal therapies may be appropriate depending on the needs of the patient, however, further studies should be conducted in order to assess their efficacy and safety in patients especially with congenital fibrinogen disorders.

### 2.2. Factor V Deficiency

Factor V (FV) deficiency is an uncommon disorder of coagulation, with a prevalence rate of one in one million people. AUB is a common finding among women with FV deficiency [17]. Paul Owren initially described this condition in a Norwegian woman with a history of bleeding including menorrhagia and epistaxis throughout her life, and prolonged prothrombin time. Historically, it has been termed Owren’s disease after him, as well as parahemophilia, due to the clinical resemblance to the classic hemophilia [6].

Factor V synthesis happens both in the liver and megakaryocytes and its half-life is 12–15 h. It is converted to its activated form, Va, following several proteolytic cleavages by thrombin or factor Xa. Along with factor Xa, it forms the prothrombinase complex, which catalyzes the conversion of prothrombin to thrombin. A defect in the activity of Factor V leads to inability to convert prothrombin to thrombin, thus conferring an increased hemorrhagic risk [17].

FV deficiency follows an autosomal recessive pattern of inheritance, although cases of acquired deficiency have been reported [27]. There are two types of FV deficiencies. In type I, which is the most form, there is concomitant deficiency of factor V antigen and activity, while in type II, which affects approximately 25% of patients, the antigen levels are normal, hence indicating pathologic protein activity. In heterozygotes, the FV activity levels are within 25–60% of normal, and they are usually asymptomatic. In homozygotes, on the other hand, FV activity levels are less than 10%, and they are more often symptomatic [17].

Gynecologic problems in women with FV deficiency include menorrhagia, ovulation-induced hemoperitoneum, and postpartum hemorrhage. Additional clinical findings that should be searched for in the patient’s history include ecchymoses, epistaxis, gingival bleeding, mucosal bleeding, GI bleeding, and hemorrhage after minor trauma. Cases of life-threatening hemorrhagic events following trauma, as well as cerebral hemorrhage, have been reported [17]. Clinical manifestations in individuals with homozygous Factor V deficiency typically occur very early in life. There seems to be no exact correlation between remaining Factor V levels and bleeding phenotype, and women with very low FV levels may still have mild to no bleeding symptoms. However, life-threatening episodes are highly indicative of severe FV deficiency [6]. PT and PTT may be prolonged but correct after mixing [10]. The definite diagnosis of Clotting Factor deficiencies relies on molecular testing [17].

Factor V deficiency should be suspected in females with AUB especially in the presence of a history of petechial hemorrhages, non-traumatic bruises, and ecchymoses, and when more common causes of disordered coagulation have been excluded. The diagnosis of FV deficiency is based on clinical features and family history, as well as routine and specified laboratory investigations. In many cases, the diagnosis is incidental, following routine laboratory workups. Prolonged PT and aPTT are suggestive of deficiency in one or more coagulation factors of the coagulation cascade common pathway. In cases of severe FV deficiency with decreased platelet FV level, the BT may also be increased. For confirmation, an FV activity assay can be performed. In the case of low FV activity, mixing studies should be conducted to distinguish between congenital deficiency and deficiency due to an inhibitor against FV. FVIII levels should also be measured to assess for combined FV and FVIII deficiency [6].

There are no well-established guidelines for treating individuals with FV deficiency. A commonly used approach is on-demand Fresh Frozen Plasma (FFP) administration, aiming to keep FV levels above 20%. Platelet transfusion is another option with some benefits over FFP. Platelet FV has greater procoagulant activity than plasma FV factor because it is stored in platelet α-granules in preactivated form. Another benefit is that platelets specifically reach the site of vascular injury, where they release the high concentrations of FV from α-granules, hence mediating local thrombin formation. On the contrary, plasma FV is susceptible to rapid neutralization from allo- or auto-antibodies, leading to suboptimal treatment results. Finally, a promising therapeutic option is a plasma-derived FV concentrate, which is currently undergoing preliminary testing [6]. For women presenting with Heavy Menstrual Bleeding (HMB) and FV deficiency as the underlying cause, episodes are usually well managed with FFP, antifibrinolytics, desmopressin, and oral contraceptives. Although FV concentrate is not currently commercially available, an in vitro study has shown its ability to fully normalize both PT and aPTT, as well as thrombin generation parameters. For women with severe FV deficiency and HMB that is refractory to standard treatment, Kaya et al. have proposed the administration of recombinant Factor VIIa (rFVIIa) as a potential therapeutic option [28].

### 2.3. Combined Factor V and Factor VIII Deficiency

Factor V and Factor VIII (F5F8D) deficiency is an extremely uncommon bleeding disorder with less than 200 cases reported in the literature so far. In a systematic review by Spiliopoulos and Kadir about Congenital F5F8D in women, only 10 case series and 15 case reports from 1976 to 2015 were recovered from the literature, accounting for a total of 86 women [8]. Oeri et al. initially described this disorder in 1954. It is estimated to affect one person per million and the prevalence is higher in countries where consanguineous marriage is common, notably in Israel, Iran, and other Middle-Eastern countries, as well as Mediterranean and South African countries [29].

It follows an autosomal recessive pattern of inheritance, and it is caused by mutations in one of two different genes–Lectin Mannose Binding Protein (LMAN1) and Multiple Coagulation Factor Deficiency 2 (MCFD2) genes–in chromosomes 18 (2p21) and 2 (2p16.3), respectively [30].

Individuals with F5F8 deficiency have low levels of both FV and FVIII, mild-to-moderate bleeding tendency, normal platelet count, and prolonged PT and aPTT tests. The usual range for FV and FVIII levels are within 5–30 U/dL of normal, although cases with levels of less that 1 U/dL or close to 50 U/dL have been noted [8]. As mentioned above, in women with FV deficiency, FVIII levels should also be measured to assess for combined deficiency.

The clinical presentation and bleeding symptoms are variable and similar to those of isolated FV or FVIII deficiency. Menorrhagia is among the main clinical presentations, along with epistaxis and excessive bleeding following trauma or surgery. In the systematic review by Spiliopoulos and Kadir, 42 (49%) women with F5F8D reported HMB, making it the most commonly reported symptom. Another serious risk for these women was recurrent and severe ovulation bleeding [29].

Successful management of menorrhagia in women with F5F8D requires a source for both FV and FVIII. FFP can replenish both factor V and factor VIII, although it is more effective in normalizing factor V levels. Various methods can restore FVIII, but currently the most common is desmopressin [8,29]. 1-desamino-8-D-arginine vasopressin (DDAVP) has been experimentally shown to increase FVIII levels in patients with F5F8D but has no effect on FV levels. It is an attractive alternative to FVIII concentrate, as it possesses the advantage of a lower cost, which is important, especially in low-income settings where F5F8D is most prevalent. Furthermore, as a synthetic drug, DDAVP lacks the potential to cause blood-borne infections, which, although occurring uncommonly thanks to the screening of donors, have been observed with FVIII concentrate administration. However, it should be noted that to date, DDAVP has been mainly used to manage bleeding symptoms and not as a preventative measure in stable patients [31].

### 2.4. Factor VII Deficiency

Factor VII deficiency initially was described in 1951 and it constitutes 28–36% of Rare Coagulation Factor Disorders making it the most common among them [11]. It is estimated that 1:300,000–500,000 have severe homozygous deficiency, while 1:350 are heterozygotes [32]. It follows an autosomal recessive inheritance pattern with variable penetrance [32].

Factor VII (FVII) is one of the vitamin K-dependent procoagulant factors. It is synthesized in the liver and excreted in plasma. When active, FVIIa initiates blood coagulation. Following vascular injury, circulating blood comes in contact with cells expressing Tissue Factor (TF), which activates blood coagulation. A TF-FVIIa complex is then formed, which leads to the activation of FX (and FXI) and FXa which then mediates prothrombin to thrombin conversion [18].

Regarding the clinical presentation of individuals with FVII deficiency, it can be variable, from asymptomatic or mildly symptomatic cases–for example easy bruising, gum bleeding, and epistaxis–to severe cerebral or gastrointestinal hemorrhage [33]. The factor levels cannot predict the clinical phenotype and severity of symptoms. Heterozygotes can present with severe hemorrhage and vice versa, homozygotes or compound homozygotes may be asymptomatic. Even homozygotes having the same mutation may still have different clinical presentations. The typical FVII level is less than 10% for homozygotes and 20–60% for heterozygotes. The diagnosis can be made through personal and family history, accompanied by an isolated prolonged PT and low factor VII level [32]. The normal range is around 50–150 IU/dL, varying according to the methods and reagents.

A recent systematic review about FVII deficiency and gynecological or obstetric issues in women was carried out by Rezan Abdul-Kadir and Keith Gomez and included 114 women from 62 publications. According to their findings, HMB was the most common bleeding symptom (82%), followed by epistaxis (21%). Hospitalization and urgent interventions for acute heavy menstrual bleeding events were needed in 14% of the cases [19].

Management of HMB in patients with FVII deficiency varies due to a lack of treatment guidelines. In the same systematic review mentioned above, treatment data were provided for forty-one out of ninety-four women with HMB and FVII deficiency. Ten out of sixteen women who were admitted acutely with HMB received blood transfusions, six required surgical intervention, and four required both. Other therapeutic interventions included factor replacement (rFVIIa, plasma-derived FVII concentrate), fresh frozen plasma (FFP), methylergobrevin, antifibrinolytics such as oral tranexamic acid, intravenous prothrombin complex, and hormonal treatment (including combined oral contraceptives, progestogen-only treatment, hormonal intrauterine devices, and a gonadotropin-releasing hormone analogue). Finally, a combination of hemostatic and hormonal treatments was used [19].

As far as heterozygotes of congenital FVII deficiency are concerned, a study of 84 heterozygotes that were paired with unaffected family members with normal FVII levels for a period of over 20 years showed no statistically significant difference between bleeding manifestations in the two groups, implying that heterozygote carriers of FVII mutations may not be at increased risk for HMB [34].

### 2.5. FX Deficiency

Factor X deficiency (FXD) affects approximately 1 in 1,000,000 people. It is an autosomal recessive disorder, and the prevalence of the heterozygote state is estimated at 1 in 500 people [20]. Like other coagulation factor deficiencies, it is most prevalent in communities where consanguinity is common.

Factor X (FX) is produced in the liver in the form of a prepropeptide which undergoes two cleavages to reach its active form. Along with factors II, VII, IX and proteins C and S, FX it is a vitamin K-dependent coagulation protein, with an important role in the coagulation pathway. It is activated by FIXa and its cofactor or by a FVIIa-tissue factor complex in the first step of the common pathway, and its product, FXa, activates prothrombin [20].

The clinical presentations of patients with FXD depend on the severity of the deficit. In mild deficiency (FX activity 0.05–0.1 IU/mL), the clinical presentation is usually asymptomatic or mild mucocutanous bleeding. In severe deficiency (FX activity < 0.01 IU/mL), there is a risk for life-threatening bleeding, including umbilical cord, intracranial hemorrhage, haemoperitoneum during ovulation, and haemarthrosis. In the mid-range (FX activity 0.01–0.05 IU/mL), these manifestations are uncommon. Heterozygote carriers with FX activity 0.1–0.3 IU/mL may experience minor mucocutaneous hemorrhages associated with surgery or trauma [7].

In a systematic review by Spiliopoulos et al. covering the years between 1960 and 2018, 332 women with Factor X deficiency were identified in the literature. The results showed that 77 out of the 332 women (23.2%) had severe deficiency (FX coagulant activity <0.01 IU/mL), plus 107 women (32.2%) had mild or moderate deficiency (>0.01 IU/mL), while in the rest the FX level was not mentioned. HMB was a relatively common presentation in these women, with an incidence rate ranging from 25% to 47% in women with a thorough bleeding history evaluation. Apart from HMB, other manifestations were reported including epistaxis, gum bleeding, haemarthrosis, bruising, GI bleeding, muscle haematoma, CNS bleeding, haematuria, and umbilical cord bleeding [7].

Blood products transfusions (FFP’s, RBC’s) and PCC administration were used to treat bleeding episodes in these patients. Other interventions included tranexamic acid, combined oral contraception (COC), progestins, and danazol. Eight women (2.4%) had haemoperitoneum from ovulation bleeding/ruptured haemorrhagic cysts and they all required blood transfusion, while six of them (75%) required surgical management (laparoscopy) [7].

Regarding the management of FXD, a consensus for treatment guidelines is currently lacking. However, current evidence supports the administration of single-factor replacement therapy with plasma-derived FX (pdFX) [35]. A 2022 review on hemophilia by Payne et al. summarizing evidence from three pharmacokinetic studies (TEN01, TEN02, TEN05) and real-life evidence from case reports and case series, provides specific suggestions regarding the administration and dosage of plasma-derived FX, as well as the monitoring of FX levels [36]. Other options when pdFX is not available include FFP, cryoprecipitate, and prothrombin complex concentrates (PCCs) [35].

### 2.6. FXI Deficiency

Factor XI (FXI) deficiency or hemophilia C or Rosenthal disease is an autosomally inherited coagulopathy which was first reported in a Jewish family in the USA. The name “hemophilia C” was initially attributed to it, in order to distinguish between hemophilia A and B, as in contrast to these well-described disorders, it affected both sexes and did not cause spontaneous bleeding with the same frequency. It is one of the most common genetic disorders, as the heterozygous frequency of FXI deficiency is 8% [22]. The highest prevalence is noted in Ashkenazi Jews. The severe form of FXI deficiency, which is characterized by FXI levels below 20%, affects approximately 1 in 1,000,000 people, and usually concerns homozygotes or compound heterozygotes. On the contrary, in heterozygotes FXI levels are 20–60% [21,37]. As mentioned above, the worldwide prevalence of RCFDs has been estimated to be between 3% and 8%. Out of those, FXI is thought to account for 23–32% [11]. Regarding the incidence of FXI deficiency among women with HMB, in a 1998 study by Kadir et al. [38], the prevalence of FXI deficiency in patients with objectively confirmed menorrhagia was 4% (with a 95% confidence interval from 1.5–8.5%).

FXI is a protein-zymogen primarily produced in the liver and secreted into the bloodstream, although small quantities can be identified in other cells such as platelets. Activators of FXI into its enzymatic form, FXIa, are FXIIa and thrombin, as well as FXIa itself (in the presence of polyanions). The activated enzyme FXIa then proceeds to activate FIX to FIXa, thus promoting the intrinsic cascade of coagulation with the ultimate goal of producing a fibrin clot. When FXI is deficient, hemostatic problems occur. Severe deficiency is mostly inherited in an autosomal-recessive manner. Nevertheless, there have been reported certain heterozygous genetic variants of mutated FXI molecules that by binding with the wild-type molecule form a heterodimer that inhibits its secretion into the blood stream. The result is reduced FXI activity levels [21].

The connection between FXI levels and bleeding tendency is not well understood and is demonstrably not linear. Severely deficient patients may have mild clinical phenotypes, while some heterozygotes can suffer from great hemorrhagic episodes [22]. Some of the common manifestations encountered by FXI-deficient patients are injury following trauma or surgery, epistaxis, and Abnormal Uterine Bleeding (AUB). Surgeries most commonly complicating the disease include urogenital tract and oropharyngeal tract procedures (49–67%) [21]. In one study by Kadir et al. [22], an objective assessment of menstrual loss using Pictorial Blood Assessment Chart (PBAC) scores in patients with inherited bleeding disorders including 20 women with FXI deficiency, showed that these women experienced prolonged menstrual periods in addition to excess menstrual bleeding. The prevalence of menorrhagia in patients with FXI deficiency was 59%, in comparison to 9–11% in the general public. Homozygous and heterozygous women did not differ regarding the incidence of menorrhagia, which endorses the independency between factor FXI levels and bleeding phenotype in patients with FXI deficiency.

Diagnostic evaluation for FXI deficiency, other than a thorough medical history, should include PT and aPTT measurements. The disease typically causes prolongation of aPTT with normal PT time. However, this may not be the case when FXI activity is above 30%, although such patients can have a positive bleeding history. Normal aPTT can only exclude severe FXI deficiency but not a mild or moderate form of the disorder. Therefore, FXI activity should be tested in the likelihood of FXI deficiency, even in the absence of an aPTT prolongation [21].

Although most people with FXI deficiency never need a therapeutic intervention, the treatment of choice is tranexamic acid. Oral combined contraceptives are also effective in limiting blood loss because of their activity on the endometrium which causes regular shedding of a thinner endometrium [22]. Replacement therapy includes FXI concentrates and recombinant factor VIIa [21]. When specific factor concentrates are not available, FFP can be used as an alternate source. In patients with FXI deficiency receiving replacement therapy with FXI and FVIIa, the risk of developing thrombosis should be considered [11].

It is worth noticing that the management of FXI deficiency can be quite challenging for a number of reasons: the bleeding phenotype is unpredictable because is not dependent on FXI levels, large amounts of FFP are required for adequate hemostasis, FXI concentrate is not available in all regions, and existing replacement therapies have a significant thrombotic risk [21].

### 2.7. Factor XIII Deficiency

Factor XIII (XIII) Deficiency is one of the least common RCFDs affecting about 1 in 3,000,000 people [14]. Since the original report of Factor XIII deficiency (FXIIID) in 1960, more than 500 cases have been recorded globally [9].

Factor XIII is a transglutaminase that cross-links fibrin fibers between amino acid residues, hence stabilizing a fibrin clot. In plasma, it has the form of a tetramer with two A-chains and two B-chains (A2B2), of which the A-chains contain the active component of FXIII and the B-chains serve as the carriers for the A-chains [11].

Individuals with congenital FXIII deficiency present with serious delayed spontaneous hemorrhage and normal coagulation tests. In symptomatic patients, FXIII activity is less than 1%. A notable difference to the rest of the clotting factor deficiencies is that clotting factor tests–BT, aPTT, PT, and Platelet count are normal in FXIIID. The diagnosis can be made by measuring FXIII antigen levels using enzyme-linked immunosorbent assays and/or by measuring its activity with functional methods. Genetic testing of the genes encoding FXIII A or FXIII B is also an option for more specific assessment [11].

In a systematic review by Sharief and Kadir, which included 27 case reports and 12 case series up to 2012 and 121 women with FXIII deficiency in total, AUB affected 31 (26%) of them, and it was the second most prevalent bleeding symptom. Umbilical bleeding was the most common one, affecting 33 (27%) of women. Ten women reported severe ovulation bleeding, out of which six needed surgical intervention [9]. Nevertheless, it is worth mentioning that variations in the reported incidence of AUB among women with FXIIID exist in the bibliography. As mentioned in a review of studies focusing on Iranian women, Naderi et al. [39,40] reported menorrhagia as an uncommon finding in Iranian patients with FXIIID. In the same review, Mansouritorghabeh et al. and Lak et al. estimated that 43% and 35% of women of reproductive age were affected by this complication, respectively [41,42].

The timely diagnosis of FXIIID deficiency is critical as it can have severe and life-threatening complications. Notably, cerebral haemorrhage occurs in up to 30% of patients with FXIIID and is an important cause of mortality. In as high as 80% of patients, the disorder manifests itself from the neonatal period with an umbilical stump hemorrhage. Other manifestations include soft tissue hemorrhage, haematomas, and inadequate wound healing. Specifically, women with FXIIID other than HMB are also more susceptible to miscarriage [39].

Prophylactic treatment should be initiated in patients with FXIIID promptly after diagnosis. Regarding the preferred treatment option, FXIII concentrate (fibrogammin) has been available in most hemophilia centers since 2009 [43]. Alternative options that were used previously include FFP and cryoprecipitate. Fibrogammin is considered a safe and effective treatment for patients with FXIII deficiency. In a prospective study by Rugeri et al. conducted on 213 patients (103 (48.4%) males and 110 (51.6%) with FXIIID who had at least one Intracranial Hemorrhage (ICH) episode before 2008, only one reported a major bleeding event after the onset of administration of Fibrogammin [44]. FXIIID concentrate is also the treatment of choice for women with FXIIID during gestation, although there is controversy regarding the dosage and intervals of administration [39]. Finally, recombinant factor XIII (rfXIII) has been proven to be a safe and efficacious treatment for patients with FXIIID and is gaining in popularity as a prophylaxis option [45,46].

### 2.8. Haemophilia Carriers

Hemophilia is an inherited bleeding disorder characterized by low levels of Factor VIII (FVIII) in hemophilia A or Factor IX (FIX) in hemophilia B. Both of these factors are crucial in the intrinsic pathway of coagulation. Due to the recessive X-chromosomal inheritance pattern, it is primarily men who are affected, while female relatives are often carriers of the mutation [47]. As a result, there exists a common misconception that hemophilia only impacts males and does not affect women and girls. This misconception has led to overlooking hemophilia carriers for many years. When a female hemophilia carrier experiences bleeding, healthcare providers usually pursue alternative diagnoses, causing a delay in recognizing the true diagnosis [23]. The exact prevalence of hemophilia carriers is not fully known, but it is estimated that for every male with hemophilia, there are approximately 3.0 to 5.0 potential carriers and 1.5 definite carriers [23]. Approximately one-third of hemophilia carriers or heterozygotes have factor levels in the hemophilia range (FVIII and FIX < 50 IU/dL), and about one in five individuals with mild hemophilia are female [23].

Female carriers typically have factor VIII or IX plasma concentrations that are approximately half of those found in healthy individuals, which is generally sufficient for normal hemostasis. However, carriers exhibit a wide range of clotting factor levels, ranging from very low to the upper limit of normal. This variability is partially attributed to X-inactivation or Lyonization, whereby some carriers express the non-mutated X-chromosome more dominantly, resulting in higher levels of FVIII or FIX, while others have a higher proportion of cells expressing the mutated X-chromosome, leading to low factor levels [47,48]. Various factors, such as maternal versus paternal inheritance, age, blood type, von Willebrand factor, body mass index, and C-reactive protein, have historically been suggested to influence factor activity [49]. A study comparing hemophilia carriers with healthy controls found that the median clotting factor level in carriers was 0.60 IU/mL (range, 0.05–2.19 IU/mL) compared to 1.02 IU/mL (range, 0.45–3.28 IU/mL) in non-carriers. Clotting factor levels ranging from 0.05 to 0.60 IU/mL were inversely associated with prolonged bleeding from small wounds and prolonged bleeding after tooth extraction, tonsillectomy, and surgeries [47]. Most studies investigating the bleeding tendency in hemophilia carriers have relied on self-reported bleeding, rather than direct observation, and reported symptoms include epistaxis, easy bruising, heavy menstrual bleeding (HMB), and post-procedural bleeding, with varying frequencies of each symptom across studies [49]. Hemarthrosis has also been observed [50]. However, studies have yielded contradictory results, with some showing a correlation between bleeding phenotype and factor levels, while others demonstrate that approximately one in four hemophilia carriers with normal FVIII/IX levels (>50 IU/dL) can still have an increased bleeding tendency [49,51].

To identify potential and confirmed hemophilia carriers within a family affected by the condition, it is crucial to create a detailed genetic pedigree [51]. When there is a suspicion of carrier status, laboratory confirmation is sought. It is important to note that while typical testing for hemophilia patients shows a prolonged activated partial thromboplastin time (aPTT) with a normal prothrombin time (PT) [5], most aPTT reagents may not detect mild deficiencies in coagulation factors (30–50% of normal), as they are more sensitive to severe deficiencies (10–20%). Additionally, the standard aPTT reagents are more sensitive to deficiencies in factors VIII, XI, and XII, rather than factor IX [24]. Therefore, even if the PTT values are within the normal range, potential carriership of hemophilia genes should not be excluded when suspected based on medical history. Hemostatic testing typically includes a one-stage FVIII and chromogenic FVIII assay for hemophilia A carriers or a one-stage FIX assay for hemophilia B carriers. Since FVIII is an acute phase reactant, it is recommended to repeat testing during a stable health state [23]. It is important to consider that low levels of factor VIII can also be observed in von Willebrand disease [52]. Lastly, it should be noted that hemophilia carriers can have normal factor levels, making genetic testing an additional option.

In 2021, a new nomenclature was established through an inclusive process involving hemophilia experts, patients, and the International Society on Thrombosis and Hemostasis (ISTH) community. The resulting nomenclature was based on personal bleeding history and baseline plasma FVIII/IX levels. Accordingly, hemophilia carriers are first divided according to their plasma FVIII/IX levels as being above or below the range (≥50 IU/dL and <50 IU/dL or alternatively ≥40 IU/dL and <40 IU/dL) [23,51].

1. Patients with below the range levels are then stratified by severity into three categories:
  - Severe Hemophilia (factor level < 1 IU/dL)
  - Moderate Hemophilia (factor level 1–5 IU/dL)
  - Mild Hemophilia (factor level > 5 and <40–50 IU/dL)
2. Patients with normal factor levels are stratified based on their bleeding phenotype, preferably according to their ISTHBAT score as
  - Asymptomatic Hemophilia carriers or
  - Symptomatic Hemophilia Carriers [23,51].

There are only a few studies considering the prevalence of HMB in hemophilia carriers. Members of the Global Emerging Homeostasis Panel (GEHEP) conducted a prospective, observational, cross-sectional study, which included 168 hemophilia carriers (155 hemophilia A and 13 hemophilia B) and 46 controls. Menorrhagia was the most common bleeding symptom among the carriers reported by 64% of them (108 out of 168). In comparison to the control group, hemophilia carriers had an average bleeding score of 1.4, while the control group had a score of 0.43. This difference was statistically significant (*p* < 0.00001) [50]. Likewise, in a multicenter, cross-sectional study that involved 125 obligate hemophilia carriers and 106 healthy controls, approximately 80% of the carriers experienced menorrhagia, in comparison to 60% of the controls [53]. Finally, in a cross-sectional study that included 87 women, 44 hemophilia A carriers and 43 controls, in comparison to the controls, hemophilia carriers reported a higher percentage of HMB as well as more frequent use of COCs as a result of HMB. Furthermore, carriers had significantly higher historic PBAC scores compared to controls (423 vs. 182.5, *p* = 0.018) [54].

Studies concerning the treatment of HMB especially in hemophilia carriers are quite scarce. Treatment options generally include hormonal therapies such as combined oral contraceptives, progesterone-only therapy, or long-acting reversible contraception, as well as hematologic medications such as recombinant or plasma-derived factor concentrates and antifibrinolytic medications in both hemophilia A and B carriers and (DDAVP) only in hemophilia A carriers [49]. Non-steroidal anti-inflammatory drugs (NSAIDs), commonly used for heavy menstrual bleeding (HMB) in the general population, are not recommended for patients with bleeding disorders due to their antiplatelet effects [23].

In a retrospective review of medical records from three hemophilia treatment centers (HTCs), 47 women and girls with hemophilia were included (37 with factor VIII deficiency, 10 with factor IX deficiency). Among them, 38 (81%) had reached menarche, and 14 were treated for their heavy menstrual bleeding (HMB) as documented in the medical charts. Antifibrinolytics were the most frequently prescribed medication for HMB, received by seven individuals (50%). Desmopressin was used by four (29%), FVIII concentrate by two (14%), and oral contraceptives by one (7%). Six out of the fourteen individuals (43%) received multiple treatments for HMB. Out of those six (43%) reported sufficient control of the bleeding, while eight (57%) reported that the measures were insufficient [55]. Finally, in a case series by Paulette Bryant et al. that included three hemophilia carriers of the same family with HMB as one of their main symptoms, adequate management of their symptoms required the combined use of recombinant factor VIII (Kovaltry) as well as tranexamic acid during their menses [56].

## 3. Conclusions

Heavy Menstrual Bleeding is a quite common problem for women of reproductive age. When the diagnosis is made and the underlying causes of HMB are being investigated, the PALM-COEIN system acts as a useful classification system. Each category of the PALM-COEIN system has been the focus of extensive scientific research, in order to guide clinicians towards the correct diagnosis. The “C” that stands for coagulation disorders has also been studied, but the most common disorders, especially Von Willenbrand disease, have received by far more attention. Our review focuses on secondary hemostasis (coagulation) disorders and summarizes the basic information about each coagulation factor deficiency: pathophysiology, genetics, clinical presentation, incidence of AUB, diagnosis, and treatment. Our aim was to create a useful guide for clinical practice for physicians investigating Abnormal Uterine Bleeding in cases where a hemostatic disorder—and more specifically a secondary hemostasis disorder—is suspected after more common diagnoses have been excluded. We hope that this review can serve as a roadmap for busy clinicians to familiarize themselves with coagulation factor disorders as a cause of Heavy Menstrual Bleeding and guide them towards more targeted research.

As it is a literature and not a systematic review it provides a non-exhaustive view of the topic based on the published literature, and it should not be used as a sole resource to guide diagnostic decisions or therapeutic interventions, but rather as an informative source and a reference for further reading.

## Figures and Tables

**Table 1:** Overview of Clotting Factor Disorders’ Prevalence and Frequency of Heavy Menstrual Bleeding.

Disorders	Prevalence	Frequency of HMB
Factor I/Fibrinogen Disorders	Afibrinogenemia 1:1 million people. The prevalence of dysfibrinogenemia and hypofibrinogenemia are hard to determine [12,13]	HMB is among the most common symptoms, reported in 37.5–100% of women [12,13,14,15,16]
Factor V Deficiency	1:1 million people [17]	Unknown/Probably Common [17]
Factor V and VIII Deficiency	Extremely rare (less than 200 cases reported in the literature) [8]	HMB is the most common symptom, reported in 49% (Systematic Review) [8]
Factor VII Deficiency	1:300,000–500,000 people [18]	HMB is the most common symptom, reported in 82% (Systematic Review) [19]
Factor X Deficiency	1:1 million people [20]	HMB is relatively common, reported in 25% to 47% (Systematic Review) [7]
Factor XI Deficiency	1:1 million people [21]	HMB is common, reported in 59% [22]
Factor XIII Deficiency	1:1–3 million people [9]	HMB is the second most common symptom, reported in 26% (Systematic Review) [9]
Haemophilia Carriers	Unknown	HMB is common, reported in 64–80% of carriers [23,24]

**Table 2:** Overview of studies on the incidence of Menorrhagia among women with Fibrinogen disorders.

First Author	Population	Incidence of Menorrhagia
Samin Mohsenian et al. [13]	Ten females with CFD (two with afibrinogenemia, two with hypodysfibrinogenemia, and six with dysfibrinogenemia)	60% had menorrhagia (6/10) (one with afibrinogenemia, one with hypodysfibrinogenemia, and four with dysfibrinogenemia)
Behnaz Tavasoli et al. [12]	eight female patients with CFD (seven with afibrinogenemia and one with dysfibrinogenemia)	37.5% had menorrhagia (3/8) (one with dysfibrinogenemia and two with afibrinogenemia)
M. Vijapurkar et al. [16]	Three female patients with afibrinogenemia	100% had menorrhagia (3/3)
M. Lak et al. [14]	Twenty female patients of childbearing age with afibrinogenemia	70% had menorrhagia (14/20)
S. Shetty et al. [15]	Nine female patients with CFD in the reproductive age group	100% had severe menorrhagia (9/9)

## Data Availability

Data used in this study are presented within the manuscript.
